# Regulatory Standards in Orphan Medicinal Product Designation in the EU

**DOI:** 10.3389/fmed.2021.698534

**Published:** 2021-06-25

**Authors:** Stelios Tsigkos, Segundo Mariz, Maria Elzbieta Sheean, Kristina Larsson, Armando Magrelli, Violeta Stoyanova-Beninska

**Affiliations:** ^1^Orphan Medicines Office, European Medicines Agency, Amsterdam, Netherlands; ^2^National Center for Drug Research and Evaluation, Istituto Superiore di Sanità, Rome, Italy; ^3^Committee of Orphan Medicinal Products, European Medicines Agency, Amsterdam, Netherlands; ^4^Medicines Evaluation Board, Utrecht, Netherlands

**Keywords:** orphan medicinal product, orphan designation, orphan regulation, significant benefit, COMP, market exclusivity

## Abstract

Twenty years of orphan regulation in Europe have now elapsed, with almost 2,400 orphan designated medicinal products and more than 190 orphan products authorised in the EU. Alongside the evolution in understanding of rare diseases, considerable regulatory knowledge has also been accumulated regarding the level of evidence that would support inclusion of products into the framework. This article reviews publications and regulatory documents pertaining to orphan medicinal product designation in the EU and discusses the general expectations in submitted applications as reflected in the current regulatory practise. Important elements to recommend granting a European orphan designation are the key considerations of orphan condition, medical plausibility, seriousness, and prevalence, while significant benefit is also assessed when there are authorised medicinal products for the sought indication. This review attempts to clarify the specific concepts currently used in that regard and discusses how the available data can be used to justify the criteria for designation. Moving away from theoretical expectations or assumptions, it stresses that the applications have to be complemented with nosological and epidemiological justifications pertaining to the proposed condition, as well as relevant data in specific non-clinical *in vivo* models or in affected patients to support inclusion into the orphan scheme.

## Introduction

Article 5 of Regulation (EC) No.141/2000 describes the procedure for orphan designation and removal from the Register of Orphan Medicinal Products (1). There is an initial evaluation upon submission of an application for orphan designation, and a second evaluation at the marketing authorisation stage (be it initial or variation of a marketing authorisation), for the maintenance of the previously granted designation. Given that initial designation can be granted at any stage of the development, applications for designation can be submitted on the basis of non-clinical data. In contrast, clinical data to justify the orphan criteria are expected at the time of the review of the criteria at the Marketing Authorisation stage, in order for the product to benefit from the associated incentives that include a 10-year Market Exclusivity.

## Orphan Condition

Incentivisation of medicinal product development in rare diseases is at the heart of the Orphan Regulation (1). Since its introduction in the end of 1999, more than 550 rare conditions have been designated (2). The effort to incentivise development in genuinely rare diseases may however raise a concern that non-rare diseases could benefit from the incentives after being subdivided into smaller groups of patients, each falling below the legal definition of rarity (not more than 5 in 10,000). The validity of a proposed condition for the purpose of orphan designation in the EU, is therefore a recurrent issue in the discussions of the Committee for Orphan Medicinal Products (COMP).

Contextualisation is key to understanding the concept of an orphan condition, and indeed the regulatory approach is frequently juxtaposed to non-regulatory views, such as clinical approaches based on benefit/risk considerations and proposals focusing on grades or stages of broader underlying diseases. An important first note is that the target condition should be established in the scientific discourse as a nosological entity, independently (and a priori) of any given application under evaluation.

Whereas eligible orphan conditions are generally described as “distinct medical entities,” there are certain aspects that are considered as reference points to delineate their distinctiveness (3). The following elements (4) are specifically used: (1) aetiology (which may include e.g., genetics), (2) histopathology, (3) pathophysiology, and (4) clinical characteristics also supported by (5) current internationally accepted classifications systems. While useful in describing the distinctiveness, all of the above elements are at times contestable, in that their respective content can also vary depending on the approach. For example, classification systems tend to have several hierarchical levels and overlapping entities. Consideration has therefore to be given on the level of “granularity” of the distinctiveness which could be considered acceptable for the purpose of the orphan designation. On the one hand, a fine-grained approach may eventually allow for an “orphanisation” of non-rare diseases, if splitting any common condition into endless rare “distinct medical entities” is accepted. On the other hand, an over simplified coarse-grained approach may appear to contravene the evolution of scientific knowledge, allowing for “lumping together” of several conditions that would otherwise be regarded as distinct, effectively blocking their access to the orphan incentives due to the lumped prevalence being above the threshold. In response to that recurring problem, the COMP has traditionally adopted a conservative approach. It is not uncommon in that context to designate a broadly worded disease, that would include several clinical variants or disease types. The problem of lumping (also known as umbrella conditions) or splitting of conditions (a subset which might be considered a distinct medical entity on its own merit) is often considered on a case by case basis by the COMP, while always aiming for consistency across applications.

The special case of a “justified subset” of a non-rare distinct medical entity merits a separate note. In particular, an application pertaining to a subset of a non-rare condition may be exceptionally considered if “patients in that subset present distinct and unique evaluable characteristics(s) with a plausible link to the condition and if such characteristics are essential for the medicinal product to carry out its action” (3). In this case, the proposed condition (a subset) would not be acceptable in its own right for the purpose of orphan designation (a broader condition would be considered instead), but the mechanism of action of the specific product additionally factors in the discussion. The issue is also explicitly discussed in the European Commission notice on the application of Articles 3, 5, and 7 of Regulation (EC) No 141/2000 on orphan medicinal products (5). Therein, it is stated that such subsets “will not be acceptable unless the sponsor provides solid scientific evidence that the activity of the product would not be shown on the larger population.” Therefore, a subset of a broader non-rare condition may be exceptionally designated if the above prerequisites are fulfilled.

There are however limited successful examples, from the early years after the orphan legislation came into force, where sponsors were granted a designation proposed for a rare subset of a non-rare distinct medical entity. The use of biomarkers in the definition of a condition in that regard has been particularly problematic. In 2014, EMA/COMP authors (6) published a paper highlighting the problems based on the experience and outcomes of submissions received for conditions defined by a biomarker. It was highlighted therein that both the “plausible linked to the condition” and the element of “exclusion of effects outside of the subset” presented with specific difficulties.

For completeness, there is also an exception referring to a “treatment modality” as a possible subject of orphan designation (3) and examples include haematopoietic stem cell transplantation (HSCT) and solid organ transplantation (SOT). Such exceptions allow for access to incentives irrespectively of the underlying condition that necessitated the transplantation in the first place.

## Medical Plausibility

The intention to “diagnose, prevent, or treat,” also referred to as “medical plausibility,” is de facto discussed by the COMP only at the initial orphan designation stage. This is because at the maintenance of orphan criteria at the marketing authorisation stage, a positive benefit/risk will be established by the CHMP.

At the time of orphan designation, in case preliminary efficacy observations in affected patients are not available, evidence in non-clinical *in vivo* models is expected in order to justify the medical plausibility of the proposal (3). It has generally been found that *in vitro* data alone is insufficient to support medical plausibility as the results cannot be extrapolated regarding the impact on the signs and symptoms associated with the rare condition. Overall, it has been reported that around a third of submissions will contain non-clinical *in vivo* data only and roughly two thirds will be submitted on the basis of preliminary clinical observations (often in combination with *in vivo* data) (7).

The validity of the non-clinical *in vivo* model used is of paramount importance in COMP discussions. Issues relating to “face validity” (the recapitulation of disease manifestations), “construct validity” (replication of the pathophysiology of the condition), and “predictive validity” (ability to allow for conclusions relevant for the human disease) may be considered in relevant deliberations (8–10). In case valid models of the proposed condition are not available, or the conduct of non-clinical studies in existing models was not possible due to e.g., cross-species limitations, bridging to other models or to data obtained with surrogate products may be exceptionally considered (11). The settings and results from such non-clinical studies are scrutinised by the COMP to establish medical plausibility.

In case the application contains clinical data in support of medical plausibility, these should pertain to the specific population in question, in contrast to data in other diseases or to observations in healthy volunteers. There have been cases where clinical observations even in a few patients have been sufficient to establish medical plausibility (e.g., EMA/OD/0000042029, November 2020 COMP minutes) (12). Clinical data are assessed at several levels. One important element is the concordance of the proposed intervention with the preventative, therapeutic or diagnostic scope of the indication as applied for designation. Other commonly discussed issues include the relevance of the studied population, study design, endpoints, use of controls, relevance of the outcomes, and magnitude of the reported effects including statistical considerations where appropriate. Depending on the disease area, measurable functional endpoints (such as behaviour, motor function, tumour growth, or survival) are usually expected to justify the clinical potential of the product, in contrast to merely pharmacodynamics regarding the mechanism of action. A mechanism of action in itself, as attested for example by changes in biomarkers that are not established as clinically relevant, would not suffice to justify medical plausibility.

## Seriousness of the Condition

The justification of this criterion has not been a highly contestable issue and there have only been a handful of cases when the COMP has questioned seriousness (e.g., EMA/OD/0000006314, July 2019 COMP minutes) (13). Nevertheless, two pieces of information are of particular importance and are expected in applications. The first one pertains to life-expectancy, the discussion of prognosis as well as causes of death. In cases of heterogeneous populations, separate discussions for the main groups of patients may be needed. The second element relates to a discussion of the sequelae and complications, resulting in long term morbidity and justifying the chronically debilitating nature of the disease. In all cases quantification based on recent literature is expected (3), which would formally document the seriousness of the condition.

## Number of Affected Individuals

One of the most basic prerequisites of any orphan framework is the legal definition of what is considered as rare. In the EU it is described as “*affecting not more than five in 10 thousand persons in the Community when the application is made*” (1). This definition may give rise to different interpretations stemming in turn from different foundations, while a series of regulatory documents, with the most recent one being the updated “points to consider on the estimation and reporting of prevalence” document (14) discuss how this provision is implemented in regulatory practise.

When assessing applications for orphan designation which are submitted on the basis of rarity (applications on the basis of insufficient return of investment are not discussed herein), considerations usually revolve around two elements: the definition of the condition and the consideration of its duration (15). The former is generally understood as “having been diagnosed with a disease according to current clinical criteria and comprehensively includes all stages or different degrees of severity of an underlying condition” (14). The latter “entails a dimension of time that has elapsed from a past diagnosis, which should be duly justified for each case” (14). Importantly, duration does not only have a bearing on the choice of the appropriate epidemiological index for reporting purposes but may also be used for indirect estimation of prevalence from incidence in the absence of direct data on prevalence.

In particular with regards to the interventional scope of an application (i.e., “prevention,” “diagnosis,” or “treatment”), certain regulatory conventions have also been introduced. If a product is intended for prevention or diagnosis of a condition, the estimate should refer to the number of people receiving the preventive treatment or the diagnostic test and not those affected by the condition itself (14).

With regards to the applications with a “treatment” scope, the choice of the epidemiological index will in general be the number of affected individuals at a given point in time (i.e., the time of application), but the actual choice will closely depend on the duration of the concerned condition. For conditions which can be considered as no longer affecting an individual after a year, (yearly) incidence rates rather than point prevalence data are expected to be used in view of the objectives of orphan designation. In contrast, for chronic conditions a point prevalence estimate is expected. Point prevalence in turn can be a partial or full index, and debates have ensued regarding the appropriate index in every case. In that context, the appropriate index for chronic diseases where a patient is considered as always affected by the condition, is the number of living patients with a previous diagnosis at the point in time of application (full prevalence, life-prevalence) (14).

In line with the above, the complete prevalence has been explicitly requested from sponsors applying for orphan designation in haematological malignancies (in multiple myeloma, myelodysplastic syndromes, chronic lymphocytic leukaemia, and several lymphomas), to take into account the relapsing/remitting and chronic natural course of the conditions (e.g., Blenrep orphan maintenance report) (16). In some cases, where complete prevalence figures cannot be estimated, long (e.g., 20-year) partial prevalence may serve as a credible proxy of complete prevalence estimates.

In terms of relevant regulatory history, an increase in the prevalence of haematological malignancies has been reflected over time in orphan procedures (17) and several withdrawn procedures in haematology have included prevalence questions (e.g., COMP Minutes of February 2021) (18). Moreover, there has been one case in renal cell carcinoma, where a designated product was considered as no longer targeting a rare disease at the maintenance stage based on complete prevalence data of 6.718/10,000 (COMP Minutes of July 2012) (19).

## Satisfactory Methods

The identification of “satisfactory methods” in the sought indication is a perquisite for the justification of significant benefit as it specifies the comparators against which a clinically relevant advantage or major contribution to patient care should be justified. However, this identification can be a contentious issue not least because the specific meaning of “satisfactory method” can vary (in general it is aligned to “a product being authorised”), but also because of several other factors including the discordance between therapeutic and orphan indications (with the latter usually worded more broadly), the potential consideration of non-pharmacological interventions (such as surgical treatments), the recommendations for standard of care as per relevant treatment guidelines, and the potential consideration of magistral and officinal formulas in line with the Commission Notice for sponsors (5).

Despite challenges, in the orphan regulatory practise “satisfactory methods” refer by and large to products authorised in the EU in the orphan condition as proposed for designation. This is because a marketing authorisation occurs upon a conclusion of a positive benefit/risk balance at either a Central EU level or at a Member State level; it is this positive B/R balance that determines which products are authorised and hence relevant (5). Sensu stricto, satisfactory methods can therefore be identified with reference to section 4.1 of the respective Summary of Product Characteristics (SmPC). Importantly, in orphan designation procedures, relevant products for the justification of significant benefit would comprise all medicines authorised in the sought condition. For marketing authorisation (maintenance) procedures, the final indication as agreed by CHMP and reflected in the new SmPC will also factor to determine which products are to be considered relevant for the significant benefit comparative exercise.

The consideration of non-pharmacological interventions as satisfactory methods of treatment, e.g., diet, plasmapheresis and surgery, has only been considered in a few past procedures (e.g., Cablivi orphan maintenance report) (20). Similarly, only a handful of examples of procedures referring to magistral or officinal formulas can be found in past procedures (e.g., EMA/OD/047/17, COMP minutes of July 2017) (21). This is because such considerations only apply when the above-mentioned methods of treatment can be considered “well known and safe and this is a general practise in the EU” (5). Similarly, products used off-label are not to be considered satisfactory in the orphan procedures (5).

## Significant Benefit

“Significant Benefit” (SB) is a critical element of the orphan regulatory framework, because it allows for orphan incentives to be awarded in indications where medicinal products have already been authorised. A comparative exercise vs. existing methods is needed that documents an improvement in favour of the proposed product; hence, SB issues may be transposed to specific data requirements. A traceable evolution of standards can be identified, with a gradual move from more abstract claims in the early days of the orphan regulation, such as theoretical (e.g., based on the assumed alternative/novel mechanism of action) or self-explanatory (based on assumed patient preferences), to claims supported by specific data in the sought indication demonstrating improved, broader or otherwise different beneficial effects compared to the authorised products (3, 5).

In that regard, after having confirmed the relevant comparators for the exercise of significant benefit (see relevant section of existing methods), a discussion based on the available data is expected to justify a clinically relevant advantage (such as improved efficacy or improved safety) or a major contribution to patient care (such as improved quality of life data) over those treatments. Most cases of significant benefit have been argued on the basis of improved efficacy (22), by providing evidence in non-clinical or clinical settings to support among others: effects on an aspect of the disease or patient population that is not covered by the label of the authorised medicines; improved effects on common aspects treated by the juxtaposed treatments; or by highlighting data showing add-on effects when the proposed product is added to the standard of care. On the other hand, arguments based on safety are in general difficult to consider for several reasons. Firstly, in particular at the initial designation step, the safety of a product under development can be considered unknown in early stages of development, and mature clinical data would be needed to appreciate it. Even if some clinical experience may be available, comparison of a limited safety database of a product under development vs. the safety database of already marketed products may also be seen as inequitable. Secondly, claims of improved safety should not only focus on one specific adverse effect or toxicity but rather based on an extensive safety comparison between the profiles of the juxtaposed products (e.g., Reblozyl Orphan Maintenance report) (23). Thirdly, in order to consider claims of improved safety, a comparable efficacy is generally expected as a prerequisite in the current regulatory practise.

Similarly, the consideration for claims based on major contribution to patient care (for examples on major contribution to patient care see the haemophilia discussions in the March 2016 COMP minutes)(24) can only be considered in an “all other being equal” basis, regarding efficacy and safety aspects. The requirement for a major contribution to patient care takes its stricter expression in case of a reformulation of an already authorised active substance (in the same indication) where a change of the route of administration is envisioned. In that case, clinical data and documentation of both the problem with the current route of administration itself as well as the effects of the proposed change for patients or carers, should be provided by the sponsor (5).

In order to justify significant benefit, in particular at the stage of marketing authorisation, indirect comparisons of clinical studies (instead of or in addition to direct comparisons in controlled studies), may be assessed. In such cases, comparability becomes an issue especially when the argued effects and differences are of limited magnitude. Juxtaposed studies may indeed differ in several aspects regarding for example the studied population, design, comparators, endpoints studied and statistical considerations. Therefore, in case independent studies are juxtaposed for the purpose of significant benefit, the methodology needs to take into consideration and eliminate confounding factors, in order to clarify whether the argued differences can be attributed to the interventions themselves. Importantly, adjusted comparisons may be used to mitigate these issues, and methodologies such as Matching-Adjusted Indirect Comparisons (MAIC), and Network Meta Analyses (NMA) have been used in past procedures (e.g., Evrysdi Orphan Maintenance Report, Takhzyro Orphan Maintenance report) (25, 26).

## Conclusions

The expected standards in orphan designation and maintenance of orphan status at time of MA in Europe have been crystallised in the past two decades and enshrined in regulatory documents and publications. In order to support an application for orphan designation in Europe, applications are expected for a rare disease that is already established and well-accepted in the scientific discourse. In general, data with the proposed active substance in an *in vivo* model of the target disease or in patients are expected to justify the intention to develop the product. This data should also support a favourable comparison in terms of efficacy, safety, or patient care, vs. any already authorised products. The discussions on any of the above can become particularly challenging at the time of review of criteria for designation at the marketing authorisation stage (for either an initial marketing authorisation or a subsequent extension of indication), something for which a sponsor can prepare by seeking Protocol Assistance from the EMA after designation. Compelling sponsor-generated clinical evidence, solid methodology and appropriate justifications are expected at the time of Marketing Authorisation, to justify that the orphan criteria are still met and support the incentive of 10 years Market Exclusivity as per the current regulatory framework.

## Author Contributions

ST wrote the first draft. All authors contributed to the conception and revisions of the manuscript.

## Conflict of Interest

The authors declare that the research was conducted in the absence of any commercial or financial relationships that could be construed as a potential conflict of interest.
